# What Should We Eat?

**Published:** 1909-02

**Authors:** 


### What Should We Eat?

THE question of food and drink, the proper nourishment of the body, is one of the most important that can engage the attention of physicians. And, we may add, it is also a subject that may be discussed by the laity with great propriety.

It is not always easy for a physician to obtain respectful hearing when he presents the question of diet to his patients or other audiences, because the listeners are apt to regard the rules laid down as arbitrary, and not always practicable nor even practical sometimes; but once let a magazine article, or an intelligent newspaper editorial appear and an attentive reading will result.

In the last issue of this JOURNAL (January, 1909), Dr. Henry Reed Hopkins presents the topic in a new way, and throws new light on the question of nourishing the body in proper manner. Every physician should pay attention to what Dr. Hopkins says about the mineral nutrients,—air, water, and the salts. While all may not agree with everything Dr. Hopkins asseverates yet enough is presented of value to deserve careful reading and patient following.

From the lay viewpoint many excellent articles, editorial and otherwise, have appeared recently in newspapers and magazines. Seldom have we read one that appeals to common sense in a greater degree than an editorial recently published in the *New York Tribune* (December 6, 1908,) entitled, "The Proper Things to Eat," which we take pleasure in reprinting.

THE PROPER THINGS TO EAT.

One of the questions on which expert opinion has been singularly divided relates to the amount of food required by a man to keep him in working condition. Two or three years ago rather convincing evidence was presented to show that surprisingly little would serve the purpose. To that side of the discussion a valuable contribution was made by Professor Chittenden, of Yale University. In illustration of the doctrine he cited the case of an acquaintance who for more than a year consumed much less than other persons, not only without detriment to his health, but apparently with distinct benefit. The argument was reinforced subsequently by the result of tests of a number of United States soldiers on whom Professor Chittenden was permitted to experiment. Though we are not sure that the New Haven physiologist recommended the selection of simple and possibly uninviting articles of diet, other advocates of a limited indulgence did. To some extent dietetic reformers have minimised the importance of appetite while recommending a diminished consumption.

The other party is now to the bat. One of its representatives, *American Medicine,* in its latest issue declares that the "physiological value of flavor" is not sufficiently recognised. It argues that an appeal to the palate is essential to digestion and that a craving for dainties (though of low nutritive value) is natural. It believes that the inadequacy of plain food is established by the fact that "the lower races," which subsist on plain and unvaried food, are "inefficient workmen," but give a better account of themselves on a richer diet. Possibly *American Medicine* overlooks other explanations of the differences in the quantity and quality of work performed by Europeans and most of the Asiatics. It also seems to ignore the danger that a person in eating things of which he is particularly fond may take more than is good for him. At any rate, the periodical just mentioned is disposed to regard *delicatessen* as "staples" and "luxuries" as "necessaries."

Here you have both sides of the case, and both are partly right. How to support a large family with a small income—to say nothing of loss of employment and unforseen doctors' bills—is a complicated problem which millions of fathers and mothers are called upon to solve. Close calculation of the expenditure for the table is absolutely essential to get the best result. Economy there deserves the first consideration, and pleasure must take the second place. On the other hand, even among those of modest means a certain amount of variety in their fare is certainly conducive to wellbeing, and the occasional indulgence in a "spread" even by the poor betrays a craving which may, after all, be entirely normal. Furthermore, it is the testimony of thousands who are so circumstanced that they can put their own useful activities ahead of everything else that generous living is often a help in realising this worthy ambition.

The truth is, no universal rule can be laid down regarding diet. No two persons are constituted precisely alike. One may digest so much more readily than his neighbor that he will get more nourishment from a given quantity of food. Even sipping fluids slowly and chewing solids with deliberation (both commendable practices) will not always establish equality with a person who can safely eat with rapidity. It is a notorious fact, too, that dishes which agree with one person disagree with another. Even the tastes of the same person change from time to time in an inexplicable manner. The wisest plan, then, seems to be for every intelligent person to find out for himself what agrees with him best and so far as is feasible to be a law unto himself. It is to be feared that the only ills which result from dietary mistakes do not come from overeating. There is a necessity for guarding against the excesses of abstinence as well.

